# An ontology-aware integration of clinical models, terminologies and guidelines: an exploratory study of the Scale for the Assessment and Rating of Ataxia (SARA)

**DOI:** 10.1186/s12911-017-0568-4

**Published:** 2017-12-06

**Authors:** Haitham Maarouf, María Taboada, Hadriana Rodriguez, Manuel Arias, Ángel Sesar, María Jesús Sobrido

**Affiliations:** 1 Department of Electronics & Computer Science, Campus Vida, University of Santiago de Compostela, Santiago de Compostela, Spain; 2 Department of Neurology, University Hospital of Santiago de Compostela, Santiago de Compostela, Spain; 3 Instituto de Investigación Sanitaria (IDIS), Centro de Investigación Biomédica en Red de Enfermedades Raras (CIBERER), Santiago de Compostela, Spain

**Keywords:** Rating scales, GDL, Human phenotype ontology, Clinical archetypes, SARA

## Abstract

**Background:**

Electronic rating scales represent an important resource for standardized data collection. However, the ability to exploit reasoning on rating scale data is still limited. The objective of this work is to facilitate the integration of the semantics required to automatically interpret collections of standardized clinical data. We developed an electronic prototype for the Scale of the Assessment and Rating of Ataxia (SARA), broadly used in neurology. In order to address the modeling challenges of the SARA, we propose to combine the best performances from OpenEHR clinical archetypes, guidelines and ontologies.

**Methods:**

A scaled-down version of the Human Phenotype Ontology (HPO) was built, extracting the terms that describe the SARA tests from free-text sources. This version of the HPO was then used as backbone to normalize the content of the SARA through clinical archetypes. The knowledge required to exploit reasoning on the SARA data was modeled as separate information-processing units interconnected via the defined archetypes. Each unit used the most appropriate technology to formally represent the required knowledge.

**Results:**

Based on this approach, we implemented a prototype named SARA Management System, to be used for both the assessment of cerebellar syndrome and the production of a clinical synopsis. For validation purposes, we used recorded SARA data from 28 anonymous subjects affected by Spinocerebellar Ataxia Type 36 (SCA36). When comparing the performance of our prototype with that of two independent experts, weighted kappa scores ranged from 0.62 to 0.86.

**Conclusions:**

The combination of archetypes, phenotype ontologies and electronic information-processing rules can be used to automate the extraction of relevant clinical knowledge from plain scores of rating scales. Our results reveal a substantial degree of agreement between the results achieved by an ontology-aware system and the human experts.

**Electronic supplementary material:**

The online version of this article (10.1186/s12911-017-0568-4) contains supplementary material, which is available to authorized users.

## Background

In the clinical domain, phenotypic abnormalities are defined as alterations in normal morphology (structural abnormalities such as *cerebellar atrophy*), physiology (functional abnormalities such as *incoordination of movement*), or behavior (such as *difficulty in social interactions*) [1]. Acquiring a better understanding of the phenotypic abnormalities associated with rare genetic diseases is crucial to improve the interpretation of the genetic tests, and the translation of genomic information into clinical practice [2]. Unlike genomic technology, collecting and analyzing phenotype data is not usually conducted following a standardized process. In clinical research, the process of collecting data ranges from determining the set of data to be gathered to decide the most appropriate computational representation. In general, this is a difficult, laborious and time-consuming task [3]. Phenotype annotation has a huge potential to automatically extract data from large amounts of existing patient records or controlled trials. Recently, substantial progress has been achieved in encoding phenotypes using the Human Phenotype Ontology (HPO) [4]. This ontology supplies a standardized core of human phenotypic abnormalities and the relationships between them. It is accessible online [5] and contains over 12,000 classes and 16,000 hierarchical relationships.

Electronic rating scales represent an important resource for standardized data collection, often providing primary and secondary outcome measures. While rating scales are used in all medical disciplines, they are especially relevant in specialties with a richness of complex phenotypic variables, such as neurology [6]. Clinical scales can measure the so-called latent variables, i.e., variables that can only be assessed indirectly through their manifestations. Examples of latent variables (or clinical dimensions) in neurological diseases include the quality and intensity of a tremor, the degree of gait imbalance or cognitive performance. These latent variables are assessed through a set of clinical questions (named statements or items) [7]. Each statement may have multiple ordered response options, for which an ordinal number (score) is assigned. The total score for the global clinical dimension is usually obtained adding up all individual scores for each statement. Well-known examples are the Mini-Mental State Examination [8], a 30-point survey used to measure cognitive impairment, or the Glasgow Coma Scale [9] to assess coma and impairment of consciousness. These instruments improve data quality by reducing subjectivity in phenotype descriptions, and by simplifying the design of data collection protocols in research studies. Usually, rating scales grade several clinical dimensions through different items. For instance, in addition to the movement disorder (i.e., disease state), the Unified Parkinson’s Disease Rating Scale assesses other clinical sub-dimensions (such as mental state, complications of treatment and activities of daily life) via 42 questions that provide a total score [10]. Reducing all content of a rating scale to a unique number (total score) may lead to loss of useful clinical information about the dimensions implicitly collected by the scale. Hence, inferring the patient’s phenotype components from the sub-scores would facilitate medical evaluation, report writing, and clinical decision-making. In this work, we chose to address the Scale for the Assessment and Rating of Ataxia (SARA) [11], a well-validated instrument to evaluate the presence and severity of cerebellar ataxia [12]. This scale is broadly used and has been applied by our group in a research on the spinocerebellar ataxia type 36 (SCA 36). A use case for the SARA scale is illustrated below.

Formal description of rating scales using standard clinical information models promotes computational data standardization, which is crucial for comparing results across studies [13], to integrate information among different applications and medical records, and to implement decision support systems. International consortia have developed standardized data models for clinical research and electronic health records, such as ISO 13606 [14], HL7 CDA [15], openEHR [16], NINDS CDE [3] and Intermountain Healthcare [17]. These efforts have focused on providing computable and formal specifications of clinical content, known as clinical models or archetypes. Such models also provide mechanisms to link the clinical statements to classes of some standard terminology or ontology. Both clinical archetypes and ontologies seek to structure patient information, according to the needs of clinical research and practice; however their perspectives are different. Archetypes model the information to mirror patient records. For example, the items *paraparesis* and *facial palsy* were recorded together into the archetype *Stroke Scale Neurological Assessment,* which is available on the Clinical Knowledge Manager (CKM) provided by the OpenEHR Foundation*.* Ontologies, on the other hand, aim to represent the meaning and relationships between clinical terms. For example, both *paraparesis* and *facial palsy* are represented as abnormalities of the nervous system in the HPO. However, the former is represented as an abnormality of the physiology, whereas the second one as an abnormality of the morphology. This ontological distinction cannot be reflected into the clinical archetype and however it is valuable to interpret the patient status. Thus, integrating ontologies with clinical archetypes would not only provide a static knowledge store, but also a dynamic resource to automatically infer patient phenotype and standardize data collection.

### Modeling rating scales by clinical archetypes

Braun et al. [18] modeled a rating scale for the assessment of multiple sclerosis patients, following a standard archetype development approach. On this basis, ontology mapping was delayed until the final stages of modeling. At this late stage, the effort required to map archetype terms to ontology entities is substantial [19], due in part to the large size of the ontologies [20]. Furthermore, designing clinical archetypes separately from ontologies may lead to major discrepancies in the meaning of the clinical statements. As a result, ontology mappings – key to achieve semantic interoperability among different data sources - are not common in the openly accessible archetypes.

### Exploiting reasoning on clinical archetypes

Exploiting reasoning on clinical archetypes is another challenge. The Guideline Definition Language (GDL) is a formalism to express decision support logic by a rule-based declarative strategy. Some researchers found GDL reliable for guideline compliance in acute stroke and chronic kidney disease [21, 22]. However, GDL does not provide much support for ontologies and related reasoning. An alternative is to transform clinical archetypes/models into OWL-DL (Ontology Web Language – Description Language) [23–26]. Following this approach, ontology reasoners, such as Pellet, Hermit or Fact++, can be used to check the OWL-based archetype consistency [24], and make automated reasoning over the OWL dataset. This structure provides support for interoperability of rule-based mechanisms [24, 27, 28]. However, having two independent versions of the same standard model - one in the language of the model itself (ADL-Archetype Definition Language) and the other in OWL - makes maintenance more difficult. Furthermore, procedural knowledge as the sum of the scores in a scale (or any complex calculation function) cannot be simply represented in terms of OWL. An interesting alternative proposed by Mugzach et al. [28] to perform a particular counting in OWL (named *k-of-N* counting by the authors) was to develop a plug-in meeting the specific requirements. However, different calculation functions would then require implementing specific plug-ins. Other researchers defined knowledge-intensive mappings from the data sources to openEHR archetypes [29, 30]. They distinguished between data-level and knowledge-level processing tasks. The former included calculation functions specified in the mappings and directly run on archetype data. The latter covered classification tasks defined using OWL classes with sufficient conditions. An integrated Personal Health Record is an alternative option to simplify data integration and clinical decision-making [31].

### Scale for the assessment and rating of ataxia

The SARA assesses severity of cerebellar dysfunction through the evaluation of eight items reflecting motor performance (gait, stance, sitting, speech disturbance, finger-chase test, nose-finger test, fast alternating hand movements and heel-shin test) [32]. For the last four items, upper and lower extremities are evaluated bilaterally, the mean values of both sides are calculated and added up to the scores of the first four items. The total score ranges from 0 (no ataxia) to 40 (most severe ataxia). The SARA is used in clinical studies for a more accurate evaluation of the patient’s motor performance, both globally and at the individual items. It is also useful for quantitative comparison of patients, ataxia types, disease stages and response to treatment, among other applications. One of the challenges of the SARA is the need to derive a qualitative description and patient classification with diagnostic implications from numerical scores. An automated system to solve this translation would greatly facilitate the use of the SARA – and, by extension, other scoring systems- by clinicians on both research and clinical routine settings.

#### A use case description

Let’s consider patients 1, 2 and 3, with the same total SARA score of 20 (Table 1). Just based on the total score, the three patients would be considered to be in similar clinical stage. However, their functional situation is notably different. While patient 1 scores very high for midline ataxia (which is concluded from the high values of the three first items) and can barely walk or sit unaided, patients 2 and 3 have compromised speech (item number 4) and limb coordination (from the values of the last four items). In turn, patients 2 and 3 – with similar sitting, standing and walking performance – have different degree of speech impairment (mild in patient 2, while verbal communication is impossible for patient 3). The total – and even just partial scores for limbs – also do not help differentiate the actual phenotype of patients 2 and 3, who have very different performance with their limbs (appendicular ataxia derived from the last four items). While patient 2 has significantly impaired motor coordination on both sides, patient 3 has a more asymmetrical cerebellar syndrome, with severe impairment on his left side, but only very mild involvement of his right side, which may be of enormous relevance to his functional ability if the patient is right-handed.

**Table 1:** An example scenario

Patient	1	2	3	4	5-R	5-L	6-R	6-L	7-R	7-L	8-R	8-L	Total Score
1	6	6	3	1	0	0	1	1	1	1	2	2	
Mean R-L					0		1		1		2		20
2	2	2	1	3	3	4	3	4	3	3	2	2	
Mean R-L					3.5		3.5		3		2		20
3	2	2	1	6	1	4	1	4	1	4	0	3	
Mean R-L					2.5		2.5		2.5		1.5		20

Table shows three hypothetical patients with the same value for the total SARA score. Items are represented by numbers: 1-Gait, 2-Stance, 3-Sitting, 4-Speech disturbance, 5-Finger-chase test, 6-Nose-finger test, 7-Fast alternating hand movements and 8-Heel-shin test. Upper and lower extremities are evaluated bilaterally. R represents Right and L, left. Mean R-L is the mean value of both sides

### Specific contribution

The main goal of our work was to develop an electronic rating scale in the clinical domain of the neurology representing both the content and the interpretation of the SARA, using the Electronic Health Record (EHR) standards and taking advantage of semantic web technologies. In order to facilitate ontology mapping and prevent large semantic discrepancies between clinical archetypes and ontologies, we propose a novel method based on the assumption that archetype design should be supported by ontologies in those clinical situations where the archetype contents are logically organized. On the other hand, the interpretation of the SARA required different types of information: data to be recorded (i.e., the content of the rating scale), knowledge about the meaning of terms in the scale (i.e., terminological knowledge), procedural knowledge to understand the meaning of scores (that can be easily expressed by guidelines), and ontological knowledge to deduce patient phenotypes. We chose to use a combination of GDL, openEHR clinical archetypes and ontologies to address the challenges of modeling the rating scale. The research questions addressed in this work are: (1) is the combination of GDL, openEHR clinical archetypes and ontologies suitable for the description of all knowledge covered by the SARA?, and (2) is it possible to achieve a reasonable integration of these technologies to efficiently model the rating scale?

## Methods

**Fig. 1:**
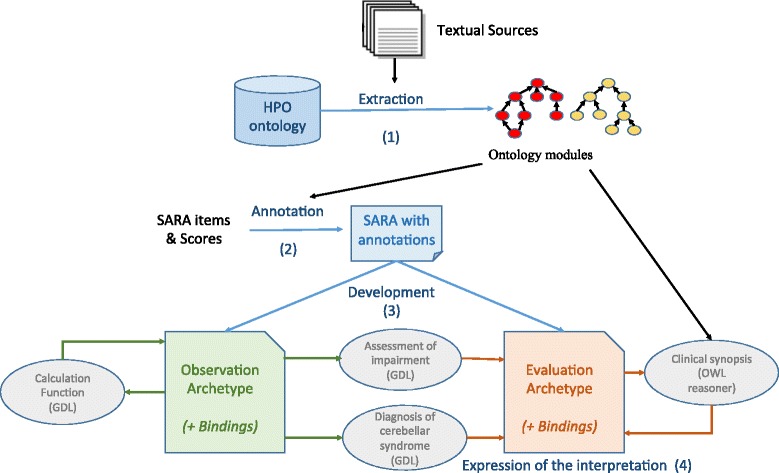
The modeling approach. It includes: 1) Extraction of a scaled-down version of the HPO; 2) Annotation of the free-text descriptions of SARA items and scores; 3) Development of two archetypes (Observation and Evaluation); 4) Definition of the information-processing units in order to express the clinical interpretation of the SARA

**Fig. 2:**
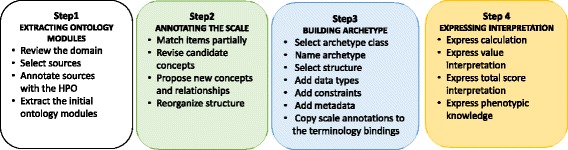
Summary of the main proposed activities for the modeling of electronic rating scales. They cover: extracting ontology modules, annotating the scale, building archetypes, and expressing interpretation

Our modeling approach is based on four main steps (Fig. 1): (1) building of a scaled-down of the HPO, through extraction of the ontology modules relevant to the SARA, 2) annotation of the free-text descriptions of the rating scale with the ontology modules, 3) development of two clinical archetypes, Observation (for normalizing the scale content) and Evaluation (for recording the clinical interpretations), and 4) Definition of the information-processing units to express the clinical interpretation support system. Each step involves several activities, described below and summarized in Fig. 2.

The modeling of SARA involved a data level - representation of the SARA items- and a knowledge level- referred to the strategy to compute the total score and interpretation of phenotype. Archetypes were used to model the data level, while GDL and OWL were used to model the knowledge level. This representation had restrictions, since openEHR models support GDL, but do not give much support for OWL and related reasoning. To bring the gap between the clinical archetypes and the ontology, mappings were defined. These mappings facilitated the translation of the archetype instances to the OWL dataset.

### Extracting the HPO ontology modules relevant to the SARA

We reviewed and collected free-text sources describing the SARA, its component tests and application. For example, the following text describes the functional subdivisions of the cerebellum in order to explain the rationale of a coordination exam: “*The cerebellum has 3 functional subdivisions, …. The first is the vestibulocerebellum. … Dysfunction of this system results in nystagmus, truncal instability (titubation), and truncal ataxia ... The following tests of the neuro exam can be divided according to which system of the cerebellum is being examined….”.* We then annotated the sources to HPO terms, using the OBO Annotator [33], a phenotype concept recognition system. In total, 12 HPO classes were selected from the annotations (Additional file 1), which provided the set of seed terms required to extracting a self-contained portion of the HPO. Such scaled-down version of the HPO covered all the classes relevant to the SARA (in total, 45 subclasses of phenotypic abnormalities). In general, self-contained portions of an ontology are referred as ontology modules/segments [34], or slims in the context of the Gene Ontology. While these modules are subgroups of a base ontology, in our case the HPO, they are also equally valid on their own [35], but simpler and more manageable than the complete ontology.

### Annotating the rating scale SARA

**Fig. 3:**
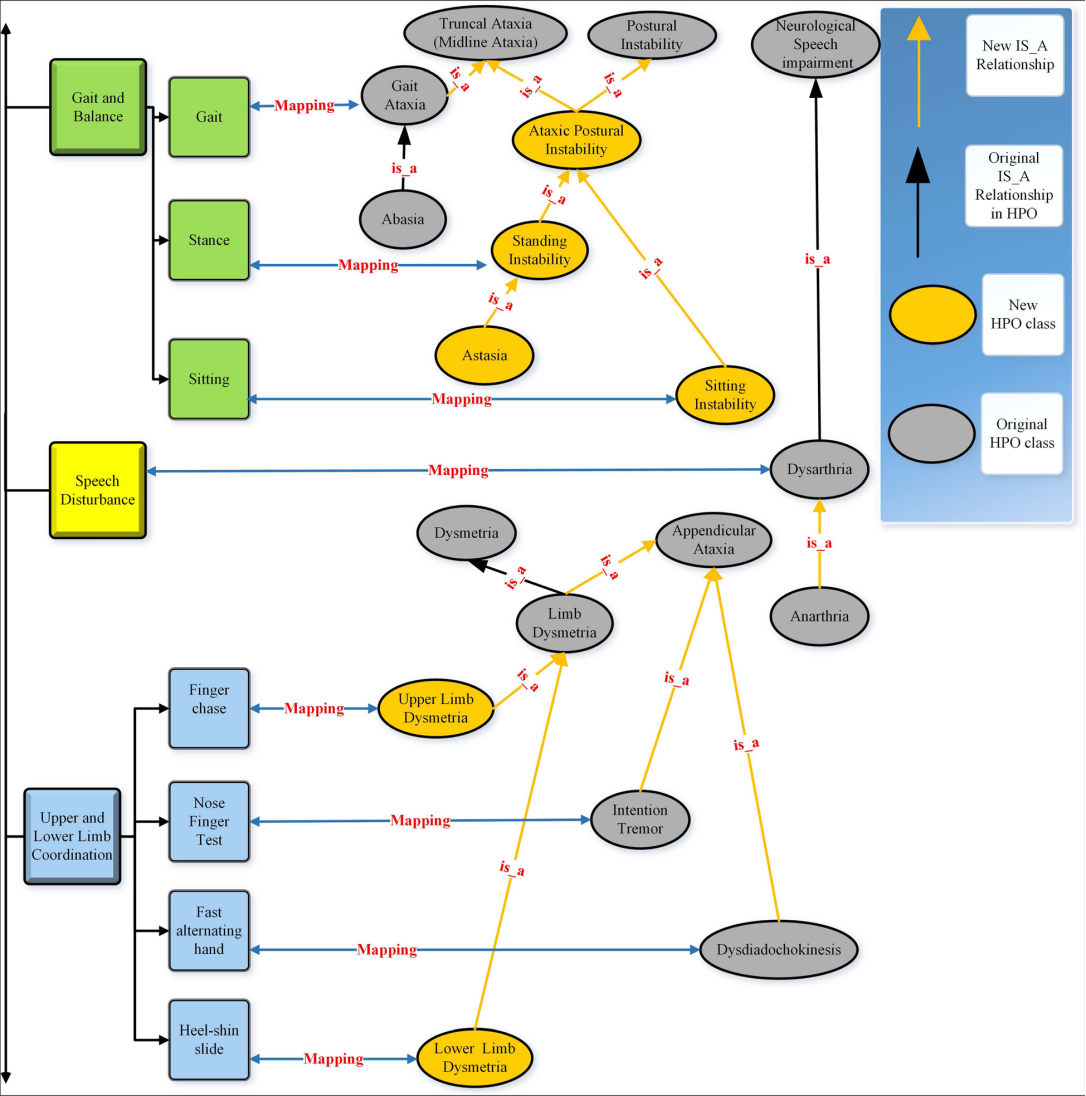
Excerpt from the set of mappings between the SARA and the ontology. Squares represent the SARA items. Gray and golden ellipses, respectively, are the original HPO classes and classes added to the ontology modules. Blue arrows are mappings between SARA items and HPO classes. Black and golden arrows, respectively, represent the original and the additional is_a relationships. Note that subsumptions in Electronic health record (Open Biological Ontologies) are represented using the is_a relationship, whereas in OWL using the subclassOf constructor

We used the scaled-down version of the HPO to annotate all items and scores of the SARA. First, we partially matched the SARA items to the ontology classes. Partial match happened when the item name was embedded inside a class name. Next, a neurologist (MJS) revised the candidate mappings, selected the most appropriate classes and proposed a minimal extension and reorganization of the scaled-down version of the HPO. Additional classes and relationships, as well as the mappings between items and classes are summarized in Fig. 3.

In order to annotate the item scores, we reused the HPO classes describing different levels of severity: borderline, mild, moderate, severe and profound. We added new subclasses based on the severity levels of their superclasses. For example, *moderate_dysarthria* was defined as a subclass of *dysarthria* with a moderate severity. Two severity scores were identified as equivalent to existing HPO phenotypes: the score 8 for the item *gait*, which was mapped to the class *abasia*; the score 6 for the item *speech disturbance*, which was mapped to *anarthria*. We also added a new class (named *astasia*) to map the score 6 to the item *stance, and* two main superclasses: i) *truncal ataxia*, which subsumed the classes *gait_ataxia* and ataxic_postural_instability (which subsumed *standing_instability* and *sitting*_instability); and ii) *appendicular ataxia*, which subsumed *limb dysmetria*, *intention tremor* and *dysdiadochokinesis*. Finally, the SARA-specific, scaled-down version of the HPO was translated to Protégé, its properties were manually modeled and the HermiT reasoner was used to check for consistency. In total, our scaled-down version of the HPO contains 109 subclasses of phenotypic abnormality (Additional file 2).

### Developing the archetypes for the SARA

For archetype design, we chose two types of clinical archetypes: *Observation* and *Evaluation*. The first was used to normalize the scale content (i.e., clinical dimensions, items and scores), whereas the second was used to record the clinical interpretations and phenotypic abnormalities derived from the achieved numerical scores. Both clinical archetypes were developed using the archetype editor provided by OpenEHR (Additional files 3 and 4).

**Fig. 4:**
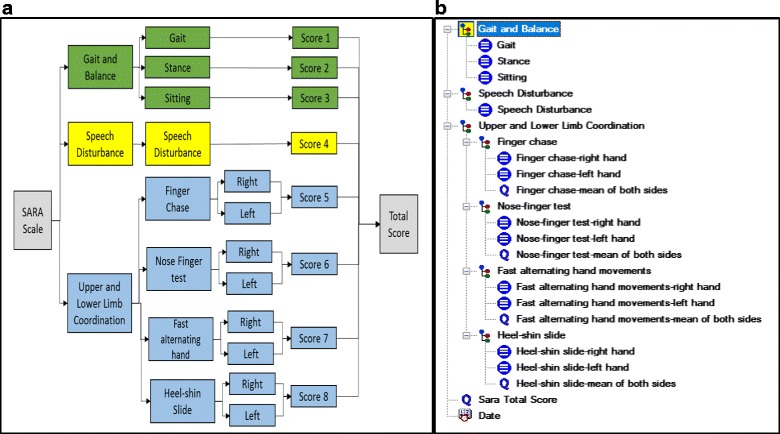
The structure of the content of the SARA. **a** The new organization of the SARA, with three main patient’s phenotype components: gait & balance, speech disturbance, and upper and lower limb coordination; **b** The clinical archetype developed for the SARA

To model the observation archetype, after adding the metadata (e.g. purpose, keywords, definition, author, etc.), we structured the content of the SARA according to this archetype, i.e., by means of a tree structure with *elements* for the three clinical dimensions (the set of items, the total score and the date of the observations), and *values* for the individual scores. Then, we defined the *elements* with proper data types, descriptions, comments, details, occurrences, constraints and possible values. We mapped each *element* to the corresponding ontology class of the scaled-down version of the HPO, using the achieved annotations (see Annotating the rating scale SARA). Finally, we structured and organized the items in accordance with the ontology, by inserting three *CLUSTER* nodes in a hierarchical structure: *i) gait and balance,* which was linked to *truncal ataxia; ii) speech disturbance,* which was linked to *dysarthria;* and iii) *upper and lower limb coordination,* which was associated with *appendicular ataxia*. This new structure did not alter the SARA, as it continued to be based on the same 8-items, but only arranged these items in a specific way (Fig. 4a). The new nodes can be viewed as clinical dimensions, but without any assigned score. Fig. 4b shows that the observation archetype meets the rating scale structure.

**Fig. 5:**
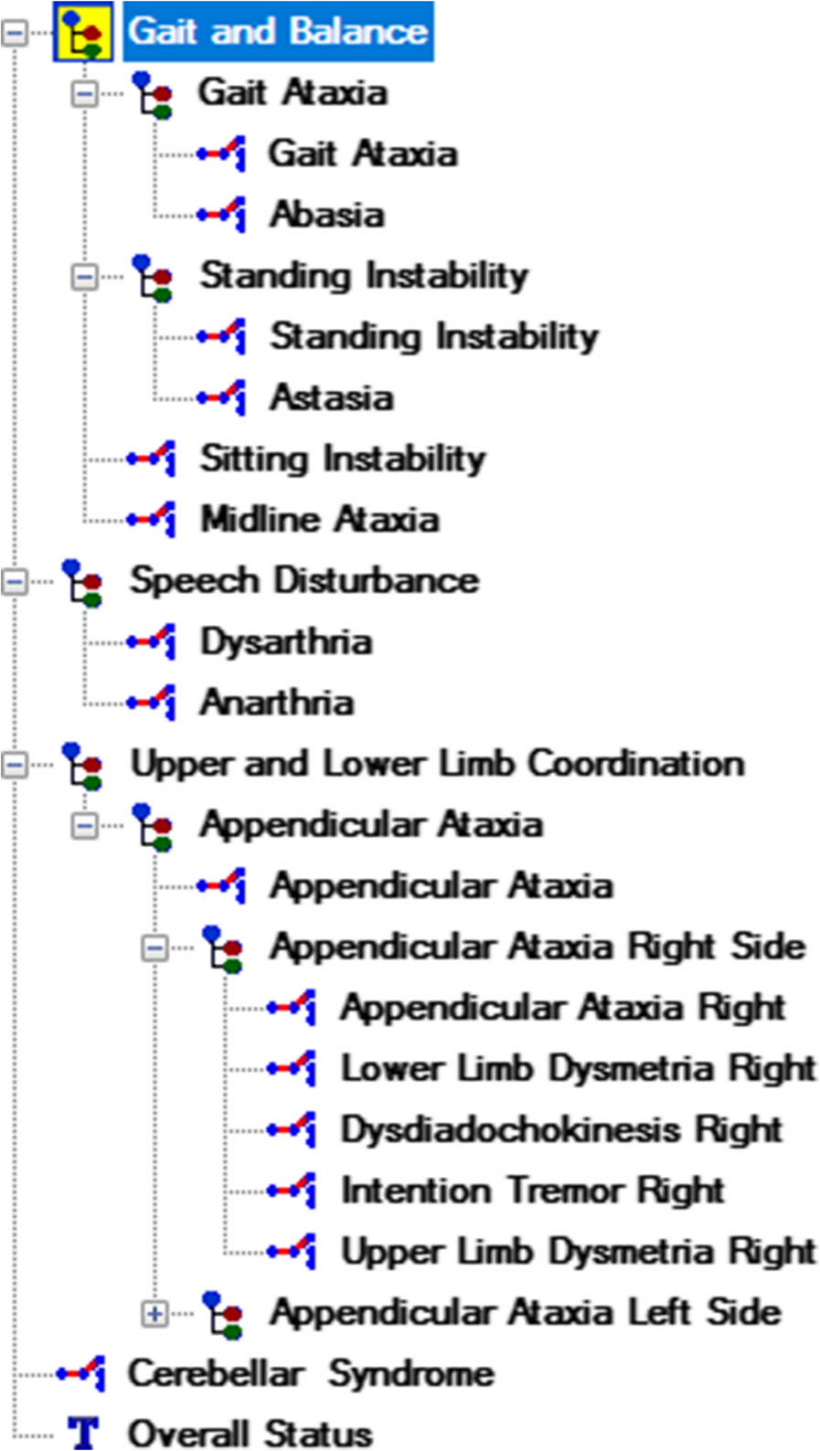
Structure of the evaluation archetype. It covers all the interpretations that can be derived from the SARA

The evaluation archetype (Fig. 5) recorded the clinical interpretations derived from the numerical scores (collected following the Observation archetype). We modeled this archetype following the same structure as the observation archetype - i.e., by means of a tree with three main clusters, one for each main clinical dimension: gait & balance, speech disturbance, and upper & lower limb coordination. Each cluster consisted of the interpretations directly or indirectly derived from the corresponding *elements* in the observation archetype. Additionally, since every *element* involved different levels of severity, so the “choice” data type was selected with Text/ Internal codes as constraints. Finally, we mapped the *elements* to ontology classes.

### Modeling the knowledge required to interpret the SARA data collection

The SARA scale supplies standardized calculation functions, but it does not provide interpretation knowledge. Thus, we analyzed the information-processing units required to automatically interpret the data obtained through the application of SARA (Table 2). With the support of the ontology and acquiring the experience of the neurologist, we were able to express the interpretation of the SARA, although this is not standardized.

**Table 2:** Information-processing units. Each unit is defined by means of the used knowledge format, the inputs, outputs and the representation language

Information-processing units	Knowledge format	Input	Output	Representation language
Calculation functions	Mathematical expressions	Observation archetype items	Observation archetype items	GDL
Assessment of the degree of impairment	Cut-off level based severity rules	Observation archetype item	Evaluation archetype items	GDL
Assessment of cerebellar syndrome	Heuristic rules	Observation archetype items	Evaluation archetype items	GDL
Clinical synopsis	Scaled-down version of the HPO	Evaluation archetype items	Evaluation archetype items	OWL

**Fig. 6:**
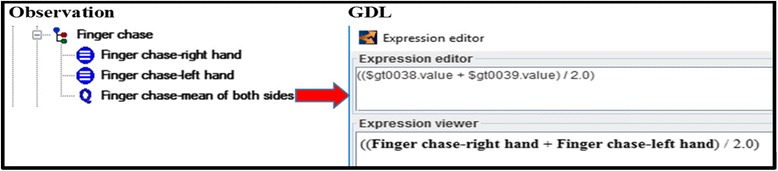
Example of a calculation function. It is expressed by a GDL rule. The function calculates the mean of an “element” defined in the observation archetype (specifically, the element finger chase). The output of the rule refers to another element of the same observation archetype

**Fig. 7:**
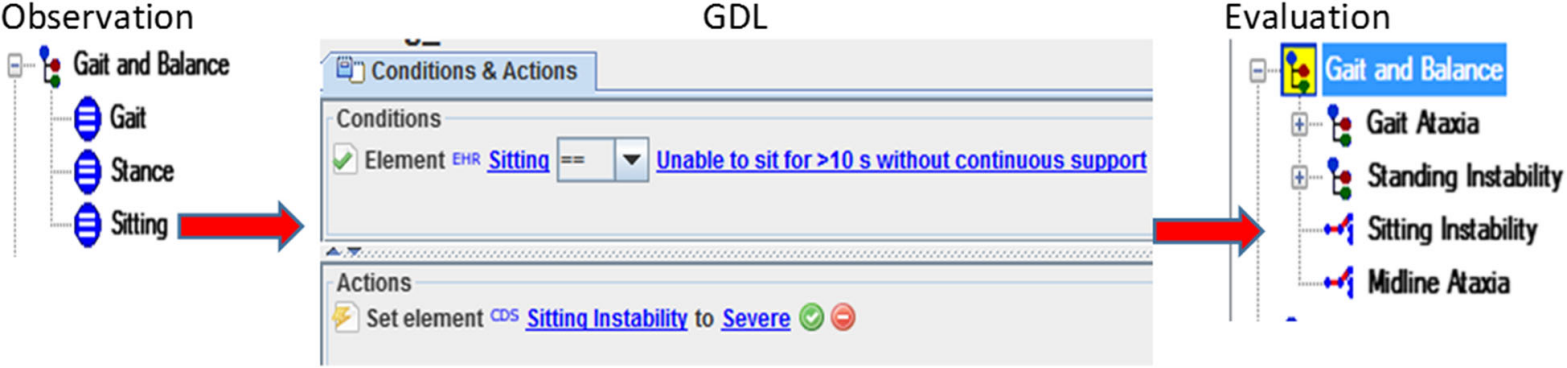
GDL rule to assess the degree of sitting instability. The rule links the score 4 for the “sitting” item to the “severe sitting instability” class

**Fig. 8:**
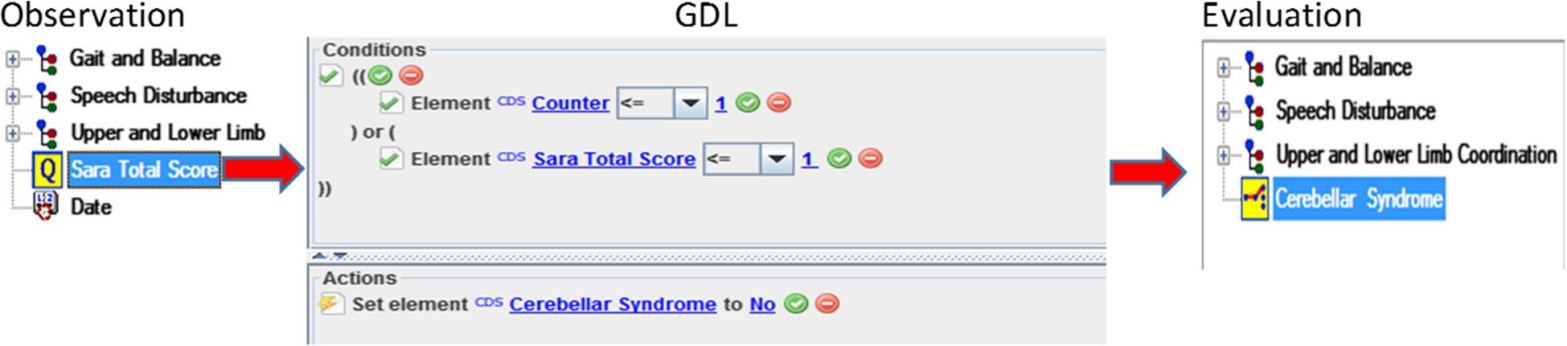
GDL rule for absence of cerebellar syndrome

The calculation functions expressed the mathematical operations to compute both the arithmetic means of the item scores and the total score. The input and output of these functions were *elements* defined in the observation archetype. In total, five GDL rules were modeled to cover these functions (Fig. 6). Cut-off levels are usually used to grade the severity of the scale items (i.e., *elements* in the observation archetype). Translating these cut-off levels to GDL required 61 rules. For example, if the score of the item *sitting* is 4, then the *sitting instability* is registered as *severe* (Fig. 7). Assessment of the cerebellar syndrome was based on the total score. The neurologist proposed several heuristic rules, which were modeled using GDL. Fig. 8 shows the rule inferring the absence of cerebellar syndrome. This knowledge was aligned with the results in [11], where the mean SARA score for controls was 0.4 ± 11.

A clinical synopsis was built to outline the phenotypic features accompanying the cerebellar syndrome. One example of such features is *midline ataxia* (*truncal ataxia*) (Fig. 5), derived from the scores of the first three SARA items and leveraging the scaled-down version of the HPO. It required using *elements* and *values* from the evaluation archetype (such as, gait ataxia, sitting and standing instability) to infer other elements and values from the same archetype using the OWL ontology. During this modeling phase, we could only simulate ontology reasoning using the Protégé tool. From the values inferred by the GDL for *elements* of the evaluation archetype, and taking into account the mappings of this archetype, we manually entered the individuals of the linked OWL classes and run the reasoner. Additional file 5 provides the rules modeled in the GDL.

## Results

### System implemented in JAVA and MySQL

**Fig. 9:**
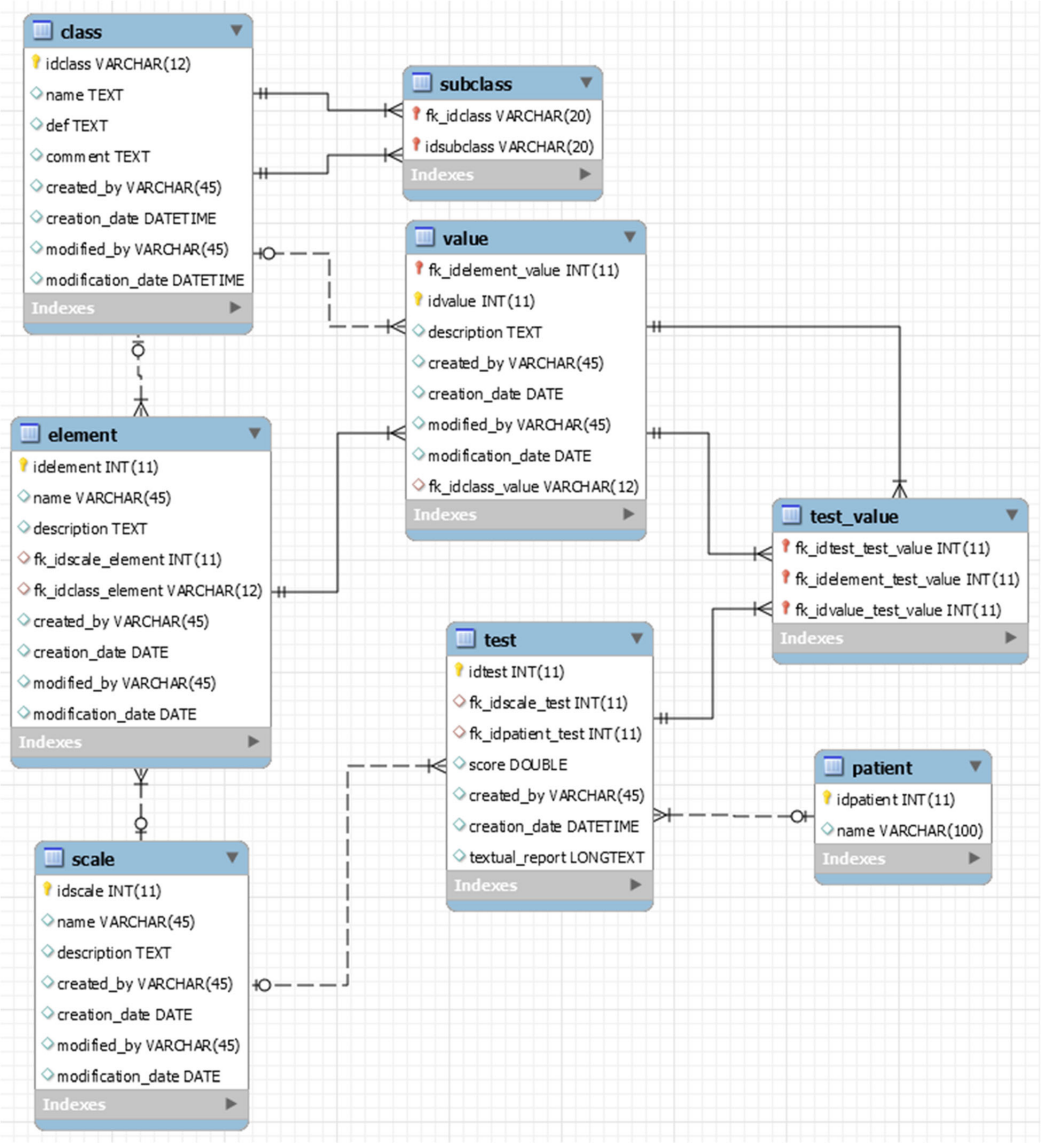
Database relational model. The table ‘patient’ recorded the patient identifier, the tables ‘test’ and ‘test_values’ stored the information about the tests covered by the SARA, and the rest of the tables recorded the information modeled in the clinical archetypes

To demonstrate the functionality of our approach, we developed a framework entitled “SMS” (SARA Management System), to be used for both the assessment of cerebellar syndrome and the production of a clinical synopsis. We used JAVA as programming language, NetBeans as integrated development environment, JAVA Swing as user interface toolkit, and MySQL as database management system. The SMS architecture was structured in three layers: “Persistence”, “Operation” and “Interface”. The persistence layer used MySQL to store the clinical archetypes, the patient input data, the data inferred by the system, and additional information. Fig. 9 shows the relational model of the database with three types of tables. The first type included the *class*, *subclass, scale, element and value* tables. All of them modeled the attributes of the clinical archetypes (*elements*, *values* and *mappings* to ontology classes). To a greater extent, they were built based on the structure of the modeled archetypes. In order to populate the database with data from the archetypes, the ADL parser provided by the OpenEHR Foundation was used to generate a dependency tree of terms and then create an XML file, which was aimed at producing archetype instances when needed. The second type of tables stored the patient data, and the last type (*test and test_value* tables) recorded the set of SARA tests.

The operation layer included all information-processing units (Table 2). The used version of GDL did not provide a Java API or any mechanism for manipulating the rules defined using the editor. Hence, the only solution was to rewrite the rules in a rule engine (such as Drools or Clips) or to implement directly in Java (we decided this second solution). However, deriving phenotype information was reasonably implemented using the OWL API [36] and the mappings linking the archetype *elements* and *values* to OWL classes. The mappings were useful to automatically create OWL individuals from the archetype instances stored into the database.

**Fig. 10:**
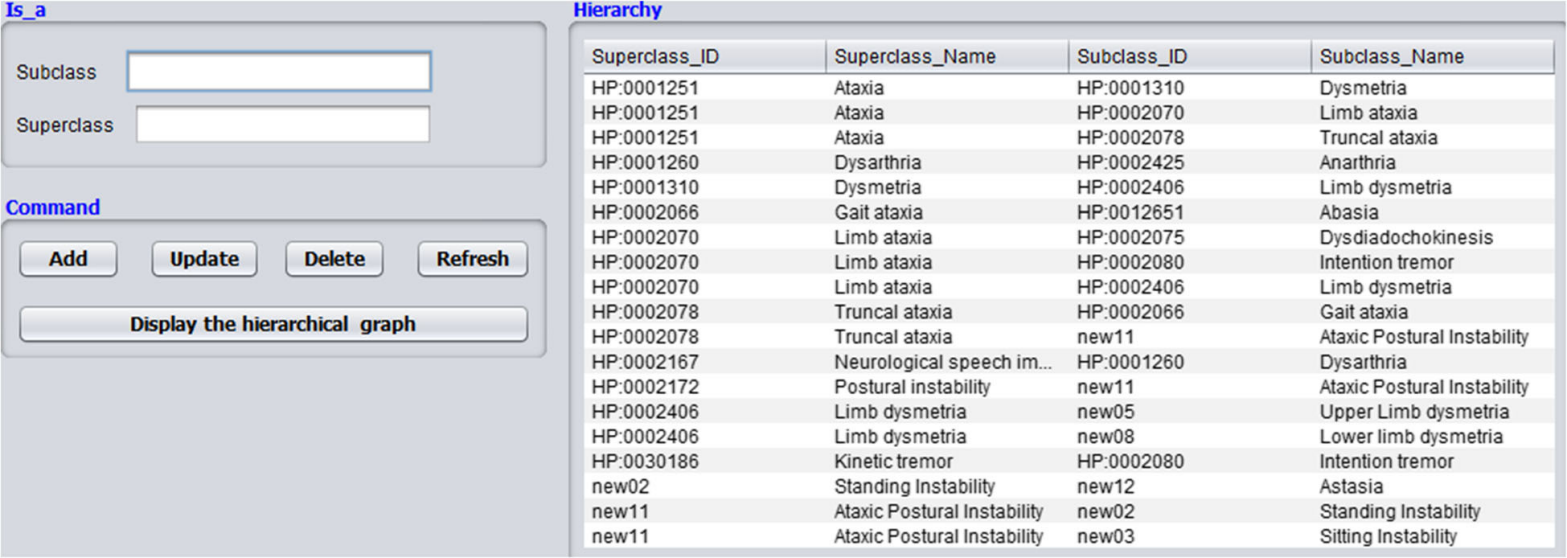
Screenshot of ontology update. The form can be used for checking, remove and update classes of the scaled-down version of the HPO used by the SMS. All the classes can be viewed in both graphical and tabular formats

**Fig. 11:**
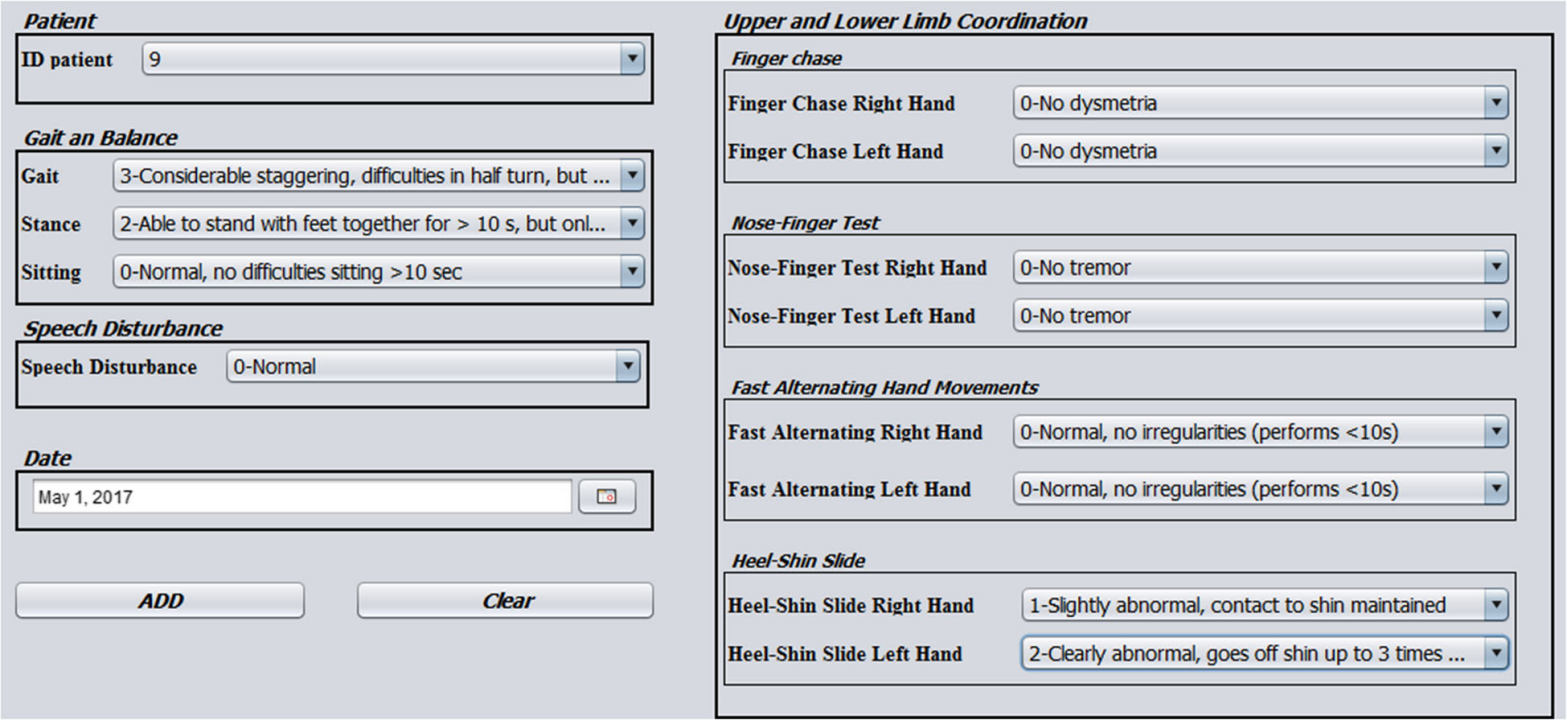
Observation form where neurologists can enter the values of the SARA scale items

**Fig. 12:**
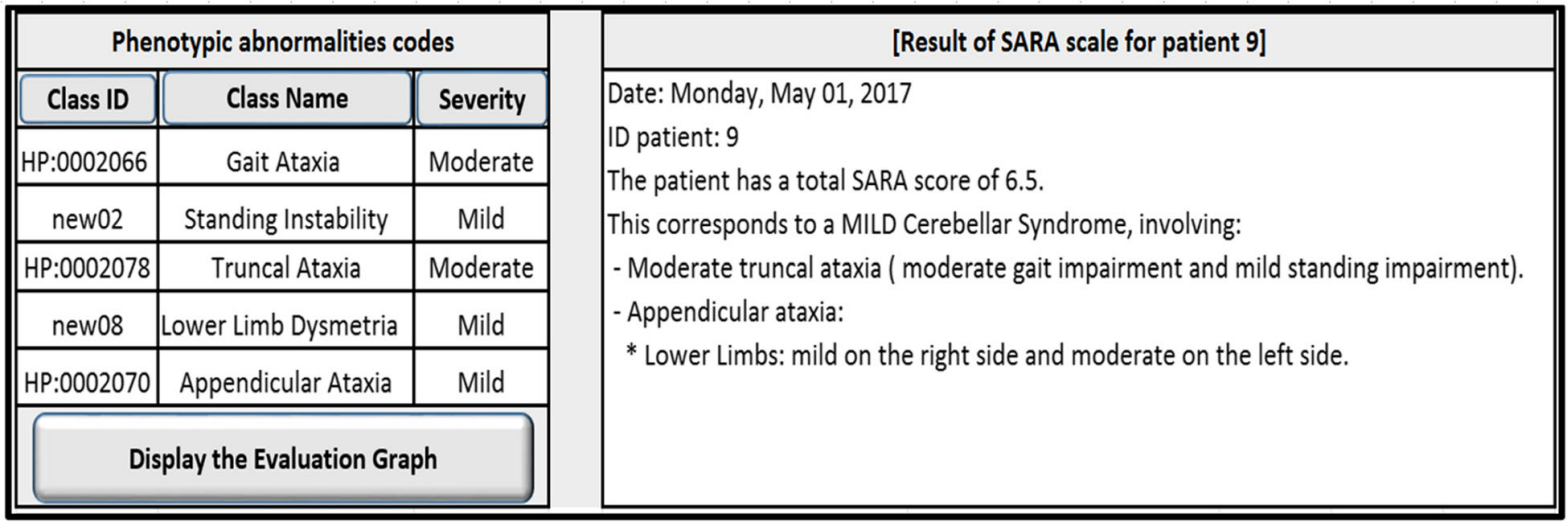
Screenshot of entry forms. It displays an example of phenotypic abnormalities derived from the data of a patient (on the left side) and a textual report summarizing the status of this patient (on the right side)

**Fig. 13:**
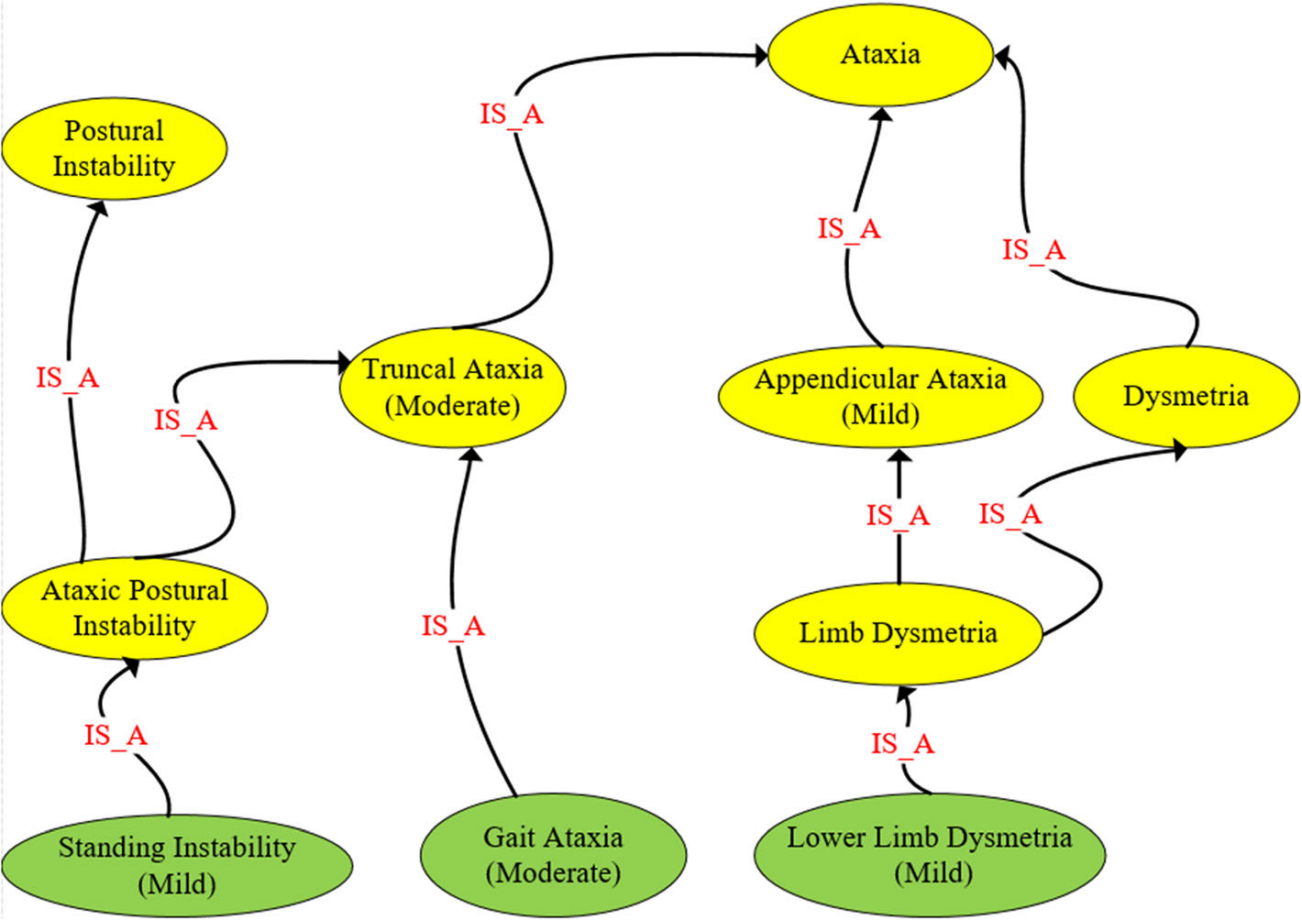
Screenshot of a graphical summary. It displays a graph visualizing the set of phenotypic abnormalities inferred by the SMS. The green ellipses represent the lower classes in the hierarchy and the yellow ellipses, the superclasses

The interface layer consisted of several input and output forms. The main form of the tool provided three menus. The first one allowed users to modify the structure of the ontology (Fig. 10), by adding, updating or deleting classes. Additionally, it provided an option to automatically generate a hierarchical graph that displayed all the available classes and their *is_a* relationships. The second menu automatically generated entry forms based on the defined archetypes (Fig. 11). These forms were used to collect patient data from the user and run the tool. The third menu displayed the outcomes (Fig. 12):

- A table with all phenotypic abnormalities inferred from the collected patient data.
- A textual report summarizing the patient status. The report included the assessment of cerebellar syndrome and a brief synopsis of the phenotypic abnormalities accompanying the syndrome.
- An evaluation graph visualizing all the patient phenotypic abnormalities (Fig. 13).

To facilitate semantic interoperable data exchange, the approach was designed to deliver the XML data collected by the SARA and also inferred by the application. Additionally, the system provided the facility to send the SARA results and the patient report by e-mail. The doctor could attach it to the patient medical record.

### Validation of the system

For validation purposes, we used anonymized data records from 28 subjects with Spinocerebellar Ataxia Type 36 (SCA36), who had been examined with the SARA scale [37]. The institutional research ethics committee approved the recruitment and study protocol, and all participants gave their written informed consent. Only the scores for each SARA item were used for this evaluation, by two independent neurologists. The validation was carried out in three steps:

1. Automatic total score calculation and interpretation: We filled out the score data for each patient to get the interpretation. The system automatically inferred 1) severity for each item in the scale, 2) assessment of cerebellar syndrome, and 3) clinical synopsis.
2. Interpretation by two independent experts in movement disorders. These neurologists analyzed the same data set blindly and independently. They described the severity of the cerebellar syndrome, truncal and appendicular ataxia from the individual scores, according to their expertise.

3) Comparison of the results obtained by the system and human experts, with a results-oriented validation perspective [38]. This perspective is based on comparing the performance of the developed tool with an expected performance provided by human experts. Among the methods to assess inter-rater agreement [39–42], we chose Weighted Kappa, as it is more compatible with ordinal scales, such as the SARA. Weighted Kappa is an inter-rater agreement measure for categorical items. It takes into account the possibility of agreements occurring by chance and it counts the disagreements differently - by weighting them. Using SPSS package, we ran Weighted Kappa test 12 times to measure the strength of agreement between the system and each neurologist, as well as between both neurologists.

Table 3 displays the Weighted Kappa values between automated and manual ratings. These values ranged between 0.62 and 0.86. In order to test whether the performance of the system reached limits considered as acceptable, we needed to define appropriate limits. Landis and Koch [43] proposed the cut-off levels shown in Table 4 to interpret kappa statistic. Table 5 shows the interpretation of the results of Table 3, following the Landis & Koch criteria.

**Table 3:** Agreement between automated and manual ratings (weighted kappa)

	System vs. 1st Neurologist	System vs. 2nd Neurologist	1st Neurologist vs. 2nd Neurologist
	Kappa value	Kappa value	Kappa value
Cerebellar syndrome	0.86	0.84	0.85
Midline Ataxia	0.80	0.84	0.86
Appendicular Ataxia (Right side)	0.71	0.80	0.86
Appendicular Ataxia (Left side)	0.62	0.78	0.84

**Table 4:** Kappa interpretation rules by Landis and Koch (1977)

Kappa Statistic	Strength of agreement
0.00	Poor
0.00-0.20	Slight
0.21-0.40	Fair
0.41-0.60	Moderate
0.61-0.80	Substantial
0.81-1.00	Almost Perfect

**Table 5:** Strength of agreement between automated and manual ratings. It follows the interpretation rules proposed by Landis and Koch

	System vs. 1st Neurologist	System vs. 2nd Neurologist	1st Neurologist vs. 2nd Neurologist
	Strength of agreement	Strength of agreement	Strength of agreement
Cerebellar syndrome	Almost Perfect	Almost Perfect	Almost Perfect
Midline Ataxia	Substantial	Almost Perfect	Almost Perfect
Appendicular Ataxia (Right)	Substantial	Substantial	Almost Perfect
Appendicular Ataxia (Left)	Substantial	Substantial	Almost Perfect

## Discussion

In this paper, a mixed method to support the development of the SARA has been presented. The method combined OpenEHR archetypes, guidelines, ontologies and reasoning. The innovation of our method rests on how these approaches were combined to get the full benefit of them. We distinguished between the modeling phase and the implementation phase. During the former, we addressed the calculation and assessment tasks by defining and executing GDL rules, and the clinical synopsis task by defining OWL classes and executing a reasoner. However, due to the lack of integration between these frameworks, we first ran the GDL framework, and then we manually entered the results in Protégé in order to infer the phenotypic abnormalities. During the implementation phase, we addressed the calculation and assessment tasks by rewriting the rules directly in Java, and the clinical synopsis task by integrating the OWL API into the system and using the mappings to create OWL individuals. We designed the approach as an archetype-based stand-alone application, providing a meaningful way for collecting and interpreting healthcare data. The application released the local EHR system of integrating the SARA, providing a standard way of delivering the collected and inferred data. Thus, the main role of this electronic rating scale was to collect the normalized data, execute the decision support logic and deliver both data and interpretations to the EHR system.

Turning to the research questions in this paper, a few conclusions can be drawn. With respect to question (1) - *Is the combination of GDL, openEHR clinical archetypes and ontologies suitable for the description of all knowledge covered by the SARA?*) -, we can conclude that a combination of OpenEHR, GDL and OWL offers a suitable framework for the purpose of describing the data and knowledge levels of the SARA. OpenEHR provides a formal specification at the data level, whereas GDL and ontologies offer formal specifications of different types of knowledge for data interpretation purpose. However, it should be emphasized that in our particular case, the knowledge level could be broken into separate information-processing units interconnected in a simple way through the two defined archetypes. However, the interpretation of a rating scale may require more complex control mechanisms, demanding more interoperability between GDL and OWL. Furthermore, the current version of GDL uses archetype data as input and output variables for all the rules, but it provides no facility to define auxiliary variables. This type of variables is sometimes necessary to model procedural knowledge, such as the counting of the scores in rating scales. At the moment, there are two solutions: 1) adding new elements to the archetype, or 2) defining an auxiliary archetype containing all the needed variables. The advantage of this second option is that leaves intact the original archetype.

The new major version of ADL includes specifications for defining explicit rules of invariant assertions, i.e., expressions that should be satisfied by all instances of an archetype. These assertions cover some calculation functions over one or several items, and also definitions of mandatory items in the presence of specific values of other items. The definition of these rules provides the same functionality as some of the GDL rules defined in our system. However, the syntax of these specifications is not stable and it is still a need of tools that offer support for the automatic handling of invariants on archetype instances. Similarly, the new ADL specification covers a section for mapping to external terminologies, which has been improved with richer mappings. Specifically, it is possible to map post-coordinated archetype codes to ontology pre-coordinated classes. This facility can be very relevant when ontology mapping is carried out in the final stages of the modeling process.

With respect to question (2) - *Is it possible to achieve a reasonable integration of these technologies to efficiently model the rating scale?* -, we showed that a full integration of these technologies to model the rating scale is not possible at the moment. In the modeling stage, the use of GDL facilitated the development and interconnection of most processing units, without resorting to external resources and encouraging knowledge sharing. We could verify and validate the SARA by testing use cases in the GDL editor. We should bear in mind that this tool automatically generates entry forms based on the defined archetypes. The forms are used to collect data from the user, run the engine and display the outcomes. However, the editor does not supply any other facility for delivering the outcomes. For example, the generation of XML instances of the archetypes would be a remarkable advance to provide the option of combining the tool with other different inference engines, such as description logic reasoners. Regarding to ontology reasoning, testing use cases based on archetypes and OWL requires tools that automate the process of converting archetype instances to OWL individuals, run the reasoner on the OWL dataset, and deliver the outcomes as instances of the archetypes.

In the implementation stage, the used version of GDL did not provide any utility to translate the modeled rules into some execution engine (e.g., drools or clips), as has been mentioned previously. Thus, this part of the implementation required substantial effort. In order to decrease the time devoted to implementation, we parsed the ADL archetypes and developed a database model based on the archetype structure. Regarding to ontology reasoning, the archetype mappings facilitated the translation of the archetype instances into OWL individuals.

The suggested approach not only focuses on the syntactic structure of the SARA, but also on leveraging a scaled-down version of the HPO from the earliest stages of the modeling of archetypes. This ontology version was a valuable resource to facilitate 1) the syntactic structure of the rating scale, 2) the terminology mapping, 3) the automated interpretation of collected data, and 4) the communication process among the information-processing units. Regarding the first point, we organized the SARA items by means of a tree structure (Fig. 5b), using the CLUSTERS class provided by OpenEHR. As mentioned above, this new organization preserved the 8-item performance of the original scale. It also differentiated the three main clinical dimensions of the SARA, although these were not assessed quantitatively. Following the OpenEHR documentation, the CLUSTERS class is provided to represent common domain patterns required in many clinical scenarios. The clinical dimensions identified into the SARA can be viewed as common domain patterns that provide a more accurate assessment of the patient’s phenotype components, clarifying the interpretation of the results. However, the observation archetype that was uploaded to the CKM, where is publicly accessible, follows the flat structure of the original rating scale. As the main goal of the CKM is to provide high-quality information models, the CKM consortium considered that a flat structure that complied with the original scale structure was more convenient. However, we think the approach presented here remains valid, as usually rating scales grade several clinical dimensions [7] and the proposed structure using CLUSTERS classes allows the proper representation of these dimensions. On the other hand, the evaluation archetype was not uploaded to the CKM, as only those archetypes that are based on some documented international assessment or very generic requirement are accepted. Following the CKM recommendations, the SARA evaluation archetype is perfectly suitable for local use.

Regarding the second point, mappings to standard vocabularies are uncommon in the clinical archetypes that are published in openly accessible repositories. In general, terminologies include a huge number of clinical terms; so manual mapping turns out to be unfeasible in practice. The extraction of the scaled-down version of the HPO provided us a means of performing terminology mapping in the earliest stages of archetype building. Just as for the clinical archetype, some parts (i.e., classes and relationships) of the scaled-down version of the HPO were reorganized to cover the SARA domain required for the ontology-driven modeling. This approach, known as ontology reuse, is an important design principle in ontologies [44, 45] that facilitates the development of specific applications.

Regarding the third point, the ontology version provided the knowledge required to infer patient phenotypic information from the data collection. For example, from the score 8 of the item *gait*, the system inferred that the patient had *abasia*, and so *gait ataxia*. However, exploiting reasoning on both ADL and ontologies is not possible at the moment. In our approach, this reasoning was needed to interpret the presence of the phenotypic abnormalities associated to the clinical dimensions of the scale. As mentioned early, a critical success factor for exploiting reasoning is the availability of ontology-based reasoning tools that use data expressed in ADL format and with capabilities to fire GDL rules. Such an integrated editor would assist with the effort at the authoring level. On the other hand, following the approaches developed in [24, 27, 28], we will transform the clinical archetypes into OWL-DL and use the ontology and rule-based mechanisms provided by Protégé to draw interpretations on data collection, with the goal of comparing the results with the ones achieved the approach developed in this work.

With regard to the interpretation of the results of our application, Table 5 reflects a very high degree of agreement between the system and the two neurologists, confirming that the approach can be a good solution to develop electronic rating scales. Even so, these excellent results should also be viewed with much caution, as the validation was carried out only with 28 patient data, all of them affected by the same rare disease (SCA36). Additionally, although the two neurologists who carried out the assessment were independent, they work in the same hospital and one of them is in the same research group as MJS, the neurologist involved in the modeling process. It therefore has to be assumed that there exists consistency between the three neurologists. Therefore, in our future work, we will evaluate the application with a larger number of patient data that are affected by diverse cerebellar ataxias, and with the help of neurologists from different hospitals. If the results are still highly satisfactory, we will develop a simple mobile application for the automatic transmission of the interpretation to the health information system. In contrast to the SMS, which was developed as a local version, the mobile application will be available for download.

Finally, although our approach was designed to implement a prototype for managing the SARA, it is rather generic and hence applicable to model other electronic rating scales, possibly in other clinical domains. To take an example, the approach could be applied to the domain of the autism spectrum disorders, which exhibit complex phenotypes affecting variables that are difficult to measure. As a consequence, standardized scales are often used to collect a large amount of phenotypic data. Recently, a phenotype ontology has been developed to identify behavioral features of importance [46]. The availability of this ontology and also the mappings to the rating scales would facilitate the implementation of prototypes like the one presented here.

## Conclusions

Nowadays, the most ataxic disorders still have no successful pharmacological therapy, and patients suffer the unavoidable degenerative disease progression. The aim of well-validated rating scales is to understand better the natural history of ataxic disorders and evaluate properly drug efficacy in clinical trials. In the present study, we focus on supporting an automatic interpretation of the SARA data collection. The work contributes to a better understanding of how clinical archetypes, guidelines and ontologies can be combined for modeling and implementing the SARA. There are several contributions in this paper. One contribution is an ontology-aware approach to modeling the two proposed clinical archetypes, which reduces the effort necessary for creating mappings and prevents large semantic discrepancies between the modeled archetypes and the ontology modules. Another contribution is the clear and explicit separation between the standard components of the scale related to the content (i.e., items, clinical dimensions and scores), which have been modeled using an *observation* archetype, and the clinical interpretations of these components, which have been normalized by an *evaluation* archetype for local use. Finally, a key contribution is the clear identification of all different types of knowledge required to interpret the data collected by the scale, and their modeling as information-processing units that communicate with one another via the two defined archetypes, providing a simple mechanism of combining ontology and rule-based reasoning.

## Additional files

Additional file 1: The seed terms returned by the OBO Annotator when the free-text sources were annotated. (TXT 282 bytes)

Additional file 2: Contains the scaled-down version of the HPO (OWL 189 kb)

Additional file 3: Contains the openEHR observation archetype related to the SARA scale. (ADL 29 kb)

Additional file 4: Contains the openEHR evaluation archetype related to the SARA scale. (ADL 27 kb)

Additional file 5: Contains the rules modeled in the GDL. (HTML 93 kb)

## Abbreviations

ADL: Archetype Definition Language
CKM: Clinical knowledge manager
EHR: Electronic health record
GDL: Guideline definition language
HL7 CDA: Health Level Seven International Clinical Document Architecture
HPO: Human phenotype ontology
ISO: International Organization for Standardization
NINDS CDE: National institute of neurological disorders and stroke common data element
OBO: Open biological ontologies
OWL-DL: Web Ontology Language – Description Language
SARA: Scale for the assessment and rating of ataxia
SCA36: Spinocerebellar Ataxia Type 36
SMS: SARA Management System
SPSS: Statistical Package for the Social Sciences
XML: Extensible Markup Language
