# Supplementary material for: An ontology-aware integration of clinical models, terminologies and guidelines: an exploratory study of the Scale for the Assessment and Rating of Ataxia (SARA)
Source: BMC Med Inform Decis Mak. 2017 Dec 6;17:159. doi: 10.1186/s12911-017-0568-4 (PMC5718136; doi:10.1186/s12911-017-0568-4)
Supplement: Supplementary file 5 — Contains the rules modeled in the GDL. (HTML 93 kb) [file 12911_2017_568_MOESM5_ESM.html]

SARA Scale GDL 


# SARA Scale GDL

## GUIDE DETAILS

|  |  |
| --- | --- |
| Description: | Scale of the Assessment and Rating of Ataxia (SARA) is a clinical scale that is based on a semiquantitative assessment of cerebellar ataxia on an impairment level. SARA has 8 items that are related to gait, stance, sitting, speech, finger-chase test, nose-finger test, fast alternating movements and heel-shin test. Although the cerebellum is directly involved in the coordination of eye movements, oculomotor functions are not considered, as the validation trials indicated that they are determined by other factors than appendicular and midline ataxia. SARA underwent a rigorous validation procedure involving three large multi-center trials in SCA and non-SCA ataxia patients, as well as controls. |
| Purpose: | The purpose of this tool is to model all the rules that are related to SARA scale to: 1. Calculate the total score  2. Interpret the total score  3. Link each scale item with its corresponding phenotypic abnormality and to determine its severity level 4. Infer the hierarchical relationships between phenotypic abnormalities |
| Use: |  |
| Misuse: |  |
| References: |  |

## AUTHOR DETAILS

|  |  |
| --- | --- |
| Name: | Haitham Maarouf |
| Email: | h\_maarouf@hotmail.com |
| Organisation: | KEAM-University of Santiago De Compostela |
| Date: | 22/04/2017 |
| Authorship lifecycle: |  |
| Copyright: |  |

## KEYWORDS

*SARA,**HPO*
  

## CONTRIBUTORS

*María Taboada ,**María Jesús Sobrido*
  

## RULE LIST

**Rule Initialization**  
**When**  
    Element **Gait severity value** does not exist  
    Element **Stance severity value** does not exist  
    Element **Sitting severity value** does not exist  
    Element **Midline severity value** does not exist  
    Element **Finger chase right severity value** does not exist  
    Element **Finger chase left severity value**  does not exist  
    Element **Nose finger test right severity value** does not exist  
    Element **Nose finger test left severity value** does not exist  
    Element **Fast alternating hand movements right severity value**  does not exist  
    Element **Fast alternating hand movements left severity value** does not exist  
    Element **Heel-shin slide right severtiy value** does not exist  
    Element **Heel-shin slide left severity value** does not exist  
    Element **Appendicular right severity value** does not exist  
    Element **Appendicular left severity value** does not exist  
    Element **Counter** does not exist  
**Then**  
    Set element **Gait severity value** to **0**  
    Set element **Stance severity value** to **0**  
    Set element **Sitting severity value** to **0**  
    Set element **Midline severity value** to **0**  
    Set element **Finger chase right severity value** to **0**  
    Set element **Finger chase left severity value**  to **0**  
    Set element **Nose finger test right severity value** to **0**  
    Set element **Nose finger test left severity value** to **0**  
    Set element **Fast alternating hand movements right severity value**  to **0**  
    Set element **Fast alternating hand movements left severity value** to **0**  
    Set element **Heel-shin slide right severtiy value** to **0**  
    Set element **Heel-shin slide left severity value** to **0**  
    Set element **Appendicular right severity value** to **0**  
    Set element **Appendicular left severity value** to **0**  
    Set element **Counter** to **0**

**Rule Counter check gait**   
**When**  
    Element **"**Gait**VALUE"** is greater than 0  
    Element **Gait** does not exist  
**Then**  
    Set element **"**Counter**MAGNITUDE"** to (**Counter** + 1)  
    Set element **Gait** to **Gait**

**Rule Counter check stance**  
**When**  
    Element **Stance** does not exist  
    Element **"**Stance**VALUE"** is greater than 0  
**Then**  
    Set element **"**Counter**MAGNITUDE"** to (**Counter** + 1)  
    Set element **Stance** to **Stance**

**Rule Counter check sitting**  
**When**  
    Element **"**Sitting**VALUE"** is greater than 0  
    Element **Sitting** does not exist  
**Then**  
    Set element **"**Counter**MAGNITUDE"** to (**Counter** + 1)  
    Set element **Sitting** to **Sitting**

**Rule Counter check speech disturbance**  
**When**  
    Element **Speech Disturbance** does not exist  
    Element **"**Speech Disturbance**VALUE"** is greater than 0  
**Then**  
    Set element **"**Counter**MAGNITUDE"** to (**Counter** + 1)  
    Set element **Speech Disturbance** to **Speech Disturbance**

**Rule Counter check finger chase right**  
**When**  
    Element **"**Finger chase-right hand**VALUE"** is greater than 0  
    Element **Finger chase-right hand** does not exist  
**Then**  
    Set element **Finger chase-right hand** to **Finger chase-right hand**  
    Set element **"**Counter**MAGNITUDE"** to (**Counter** + 1)

**Rule Counter check finger chase left**  
**When**  
    Element **Finger chase-left hand** does not exist  
    Element **"**Finger chase-left hand**VALUE"** is greater than 0  
**Then**  
    Set element **"**Counter**MAGNITUDE"** to (**Counter** + 1)  
    Set element **Finger chase-left hand** to **Finger chase-left hand**

**Rule Counter Nose finger test right**  
**When**  
    Element **Nose-finger test-right hand** does not exist  
    Element **"**Nose-finger test-right hand**VALUE"** is greater than 0  
**Then**  
    Set element **"**Counter**MAGNITUDE"** to (**Counter** + 1)  
    Set element **Nose-finger test-right hand** to **Nose-finger test-right hand**

**Rule Counter Nose finger test left**  
**When**  
    Element **Nose-finger test-left hand** does not exist  
    Element **"**Nose-finger test-left hand**VALUE"** is greater than 0  
**Then**  
    Set element **"**Counter**MAGNITUDE"** to (**Counter** + 1)  
    Set element **Nose-finger test-left hand** to **Nose-finger test-left hand**

**Rule Counter check fast alternating hand movements right**  
**When**  
    Element **Fast alternating hand movements-right hand** does not exist  
    Element **"**Fast alternating hand movements-right hand**VALUE"** is greater than 0  
**Then**  
    Set element **Fast alternating hand movements-right hand** to **Fast alternating hand movements-right hand**  
    Set element **"**Counter**MAGNITUDE"** to (**Counter** + 1)

**Rule Counter check fast alternating hand movements left**  
**When**  
    Element **Fast alternating hand movements-left hand** does not exist  
    Element **"**Fast alternating hand movements-left hand**VALUE"** is greater than 0  
**Then**  
    Set element **Fast alternating hand movements-left hand** to **Fast alternating hand movements-left hand**  
    Set element **"**Counter**MAGNITUDE"** to (**Counter** + 1)

**Rule Counter check heel shin slide right**  
**When**  
    Element **Heel-shin slide-right hand** does not exist  
    Element **"**Heel-shin slide-right hand**VALUE"** is greater than 0  
**Then**  
    Set element **Heel-shin slide-right hand** to **Heel-shin slide-right hand**  
    Set element **"**Counter**MAGNITUDE"** to (**Counter** + 1)

**Rule Counter check heel shin slide left**  
**When**  
    Element **"**Heel-shin slide-left hand**VALUE"** is greater than 0  
    Element **Heel-shin slide-left hand** does not exist  
**Then**  
    Set element **"**Counter**MAGNITUDE"** to (**Counter** + 1)  
    Set element **Heel-shin slide-left hand** to **Heel-shin slide-left hand**

**Rule Calculate finger chase mean**  
**When**  
**Then**  
    Set element **"**Finger chase-mean of both sides**MAGNITUDE"** to ((**Finger chase-right hand** + **Finger chase-left hand**) / 2.0)  
    Set element **"**Finger chase-mean of both sides**PRECISION"** to 1

**Rule Calculate nose finger test mean**  
**When**  
**Then**  
    Set element **"**Nose-finger test-mean of both sides**PRECISION"** to 1  
    Set element **"**Nose-finger test-mean of both sides**MAGNITUDE"** to ((**Nose-finger test-right hand** + **Nose-finger test-left hand**) / 2.0)

**Rule Calculate fast alternating hand movements mean**  
**When**  
**Then**  
    Set element **"**Fast alternating hand movements-mean of both sides**PRECISION"** to 1  
    Set element **"**Fast alternating hand movements-mean of both sides**MAGNITUDE"** to ((**Fast alternating hand movements-right hand** + **Fast alternating hand movements-left hand**) / 2.0)

**Rule Calculate Heel-shin mean**  
**When**  
**Then**  
    Set element **"**Heel-shin slide-mean of both sides**PRECISION"** to 1  
    Set element **"**Heel-shin slide-mean of both sides**MAGNITUDE"** to ((**Heel-shin slide-right hand** + **Heel-shin slide-left hand**) / 2.0)

**Rule Calculate total score**  
**When**  
**Then**  
    Set element **"**Sara Total Score**MAGNITUDE"** to (((((((**Gait** + **Stance**) + **Sitting**) + **Speech Disturbance**) + **Finger chase-mean of both sides**) + **Nose-finger test-mean of both sides**) + **Fast alternating hand movements-mean of both sides**) + **Heel-shin slide-mean of both sides**)  
    Set element **"**Sara Total Score**PRECISION"** to 1

**Rule Gait normal**  
**When**  
    Element **Gait** equals to **Normal, no difficulties in walking, turning and walking tandem (up to one misstep allowed)**  
**Then**  
    Set element **Gait** to **Gait**  
    Set element **Gait Ataxia** to **Normal**

**Rule Gait Borderline**  
**When**  
    Element **Gait** equals to **Slight difficulties, only visible when walking 10 consecutive steps in tandem**  
**Then**  
    Set element **"**Gait severity value**MAGNITUDE"** to 1  
    Set element **Gait Ataxia** to **Borderline**

**Rule Gait mild**  
**When**  
    Element **Gait** equals to **Clearly abnormal, tandem walking >10 steps not possible**  
**Then**  
    Set element **"**Gait severity value**MAGNITUDE"** to 2  
    Set element **Gait Ataxia** to **Mild**

**Rule Gait moderate**  
**When**  
    ((  
        Element **Gait** equals to **Considerable staggering, difficulties in half-turn, but without support**  
    )    **or** (  
        Element **Gait** equals to **Marked staggering, intermittent support of the wall required**  
    ))  
**Then**  
    Set element **"**Gait severity value**MAGNITUDE"** to 3  
    Set element **Gait Ataxia** to **Moderate**

**Rule Gait severe**  
**When**  
    ((  
        Element **Gait** equals to **Severe staggering, permanent support of one stick or light support by one arm required**  
    )    **or** (  
        ((  
            Element **Gait** equals to **Walking > 10 m only with strong support (two special sticks or stroller or accompanying person)**  
        )        **or** (  
            Element **Gait** equals to **Walking < 10 m only with strong support (two special sticks or stroller or accompanying person)**  
        ))  
    ))  
**Then**  
    Set element **Gait Ataxia** to **Severe**  
    Set element **"**Gait severity value**MAGNITUDE"** to 4

**Rule Gait profound**  
**When**  
    Element **Gait** equals to **Unable to walk, even supported**  
**Then**  
    Set element **Gait Ataxia** to **profound**  
    Set element **"**Gait severity value**MAGNITUDE"** to 5

**Rule Stance normal**  
**When**  
    Element **Stance** equals to **Normal, able to stand in tandem for > 10 s**  
**Then**  
    Set element **Stance** to **Stance**  
    Set element **Standing Instability** to **Normal**

**Rule Stance borderline**  
**When**  
    Element **Stance** equals to **Able to stand with feet together without sway, but not in tandem for > 10s**  
**Then**  
    Set element **"**Stance severity value**MAGNITUDE"** to 1  
    Set element **Standing Instability** to **Borderline**

**Rule Stance mild**  
**When**  
    ((  
        Element **Stance** equals to **Able to stand with feet together for > 10 s, but only with sway**  
    )    **or** (  
        Element **Stance** equals to **Able to stand for > 10 s without support in natural position, but not with feet together**  
    ))  
**Then**  
    Set element **"**Stance severity value**MAGNITUDE"** to 2  
    Set element **Standing Instability** to **Mild**

**Rule Stance moderate**  
**When**  
    Element **Stance** equals to **Able to stand for >10 s in natural position only with intermittent support**  
**Then**  
    Set element **"**Stance severity value**MAGNITUDE"** to 3  
    Set element **Standing Instability** to **Moderate**

**Rule Stance severe**  
**When**  
    Element **Stance** equals to **Able to stand >10 s in natural position only with constant support of one arm**  
**Then**  
    Set element **"**Stance severity value**MAGNITUDE"** to 4  
    Set element **Standing Instability** to **Severe**

**Rule Stance profound**  
**When**  
    Element **Stance** equals to **Unable to stand for >10 s even with constant support of one arm**  
**Then**  
    Set element **Standing Instability** to **profound**  
    Set element **"**Stance severity value**MAGNITUDE"** to 5

**Rule Sitting normal**  
**When**  
    Element **Sitting** equals to **Normal, no difficulties sitting >10 sec**  
**Then**  
    Set element **Sitting Instability** to **Normal**  
    Set element **Sitting** to **Sitting**

**Rule Sitting borderline**  
**When**  
    Element **Sitting** equals to **Slight difficulties, intermittent sway**  
**Then**  
    Set element **"**Sitting severity value**MAGNITUDE"** to 1  
    Set element **Sitting Instability** to **Borderline**

**Rule Sitting mild**  
**When**  
    Element **Sitting** equals to **Constant sway, but able to sit > 10 s without support**  
**Then**  
    Set element **"**Sitting severity value**MAGNITUDE"** to 2  
    Set element **Sitting Instability** to **Mild**

**Rule Sitting moderate**  
**When**  
    Element **Sitting** equals to **Able to sit for > 10 s only with intermittent support**  
**Then**  
    Set element **"**Sitting severity value**MAGNITUDE"** to 3  
    Set element **Sitting Instability** to **Moderate**

**Rule Sitting severe**  
**When**  
    Element **Sitting** equals to **Unable to sit for >10 s without continuous support**  
**Then**  
    Set element **"**Sitting severity value**MAGNITUDE"** to 4  
    Set element **Sitting Instability** to **Severe**

**Rule speech disturbance normal**  
**When**  
    Element **Speech Disturbance** equals to **Normal**  
**Then**  
    Set element **Speech Disturbance** to **Speech Disturbance**  
    Set element **Dysarthria** to **Normal**

**Rule speech disturbance borderline**  
**When**  
    Element **Speech Disturbance** equals to **Suggestion of speech disturbance**  
**Then**  
    Set element **Dysarthria** to **Borderline**

**Rule speech disturbance mild**  
**When**  
    Element **Speech Disturbance** equals to **Impaired speech, but easy to understand**  
**Then**  
    Set element **Dysarthria** to **Mild**

**Rule speech disturbance moderate**  
**When**  
    ((  
        Element **Speech Disturbance** equals to **Occasional words difficult to understand**  
    )    **or** (  
        Element **Speech Disturbance** equals to **Many words difficult to understand**  
    ))  
**Then**  
    Set element **Dysarthria** to **Moderate**

**Rule speech disturbance severe**  
**When**  
    Element **Speech Disturbance** equals to **Only single words understandable**  
**Then**  
    Set element **Dysarthria** to **Severe**

**Rule speech disturbance profound**  
**When**  
    Element **Speech Disturbance** equals to **Speech unintelligible / anarthria**  
**Then**  
    Set element **Dysarthria** to **Profound**

**Rule Finger chase right normal**  
**When**  
    Element **Finger chase-right hand** equals to **No dysmetria**  
**Then**  
    Set element **Upper Limb Dysmetria Right** to **Normal**  
    Set element **Finger chase-right hand** to **Finger chase-right hand**

**Rule Finger chase right mild**  
**When**  
    Element **Finger chase-right hand** equals to **Dysmetria, under/ overshooting target <5 cm**  
**Then**  
    Set element **"**Finger chase right severity value**MAGNITUDE"** to 2  
    Set element **Upper Limb Dysmetria Right** to **Mild**

**Rule Finger chase right moderate**  
**When**  
    Element **Finger chase-right hand** equals to **Dysmetria, under/ overshooting target < 15 cm**  
**Then**  
    Set element **"**Finger chase right severity value**MAGNITUDE"** to 3  
    Set element **Upper Limb Dysmetria Right** to **Moderate**

**Rule Finger chase right severe**  
**When**  
    ((  
        Element **Finger chase-right hand** equals to **Dysmetria, under/ overshooting target > 15 cm**  
    )    **or** (  
        Element **Finger chase-right hand** equals to **Unable to perform 5 pointing movements**  
    ))  
**Then**  
    Set element **"**Finger chase right severity value**MAGNITUDE"** to 4  
    Set element **Upper Limb Dysmetria Right** to **Severe**

**Rule Finger chase left normal**  
**When**  
    Element **Finger chase-left hand** equals to **No dysmetria**  
**Then**  
    Set element **Upper Limb Dysmetria Left** to **Normal**  
    Set element **Finger chase-left hand** to **Finger chase-left hand**

**Rule Finger chase left mild**  
**When**  
    Element **Finger chase-left hand** equals to **Dysmetria, under/ overshooting target <5 cm**  
**Then**  
    Set element **"**Finger chase left severity value** MAGNITUDE"** to 2  
    Set element **Upper Limb Dysmetria Left** to **Mild**

**Rule Finger chase left moderate**  
**When**  
    Element **Finger chase-left hand** equals to **Dysmetria, under/ overshooting target < 15 cm**  
**Then**  
    Set element **"**Finger chase left severity value** MAGNITUDE"** to 3  
    Set element **Upper Limb Dysmetria Left** to **Moderate**

**Rule Finger chase left severe**  
**When**  
    ((  
        Element **Finger chase-left hand** equals to **Dysmetria, under/ overshooting target > 15 cm**  
    )    **or** (  
        Element **Finger chase-left hand** equals to **Unable to perform 5 pointing movements**  
    ))  
**Then**  
    Set element **"**Finger chase left severity value** MAGNITUDE"** to 4  
    Set element **Upper Limb Dysmetria Left** to **Severe**

**Rule nose finger test right normal**  
**When**  
    Element **Nose-finger test-right hand** equals to **No tremor**  
**Then**  
    Set element **Intention Tremor Right** to **Normal**  
    Set element **Nose-finger test-right hand** to **Nose-finger test-right hand**

**Rule nose finger test righ mild**  
**When**  
    Element **Nose-finger test-right hand** equals to **Tremor with an amplitude < 2 cm**  
**Then**  
    Set element **"**Nose finger test right severity value**MAGNITUDE"** to 2  
    Set element **Intention Tremor Right** to **Mild**

**Rule nose finger test righ moderate**  
**When**  
    Element **Nose-finger test-right hand** equals to **Tremor with an amplitude < 5 cm**  
**Then**  
    Set element **"**Nose finger test right severity value**MAGNITUDE"** to 3  
    Set element **Intention Tremor Right** to **Moderate**

**Rule nose finger test righ severe**  
**When**  
    ((  
        Element **Nose-finger test-right hand** equals to **Tremor with an amplitude > 5 cm**  
    )    **or** (  
        Element **Nose-finger test-right hand** equals to **Unable to perform 5 pointing movements**  
    ))  
**Then**  
    Set element **"**Nose finger test right severity value**MAGNITUDE"** to 4  
    Set element **Intention Tremor Right** to **Severe**

**Rule nose finger test left normal**  
**When**  
    Element **Nose-finger test-left hand** equals to **No tremor**  
**Then**  
    Set element **Intention Tremor Left** to **Normal**  
    Set element **Nose-finger test-left hand** to **Nose-finger test-left hand**

**Rule nose finger test left mild**  
**When**  
    Element **Nose-finger test-left hand** equals to **Tremor with an amplitude < 2 cm**  
**Then**  
    Set element **"**Nose finger test left severity value**MAGNITUDE"** to 2  
    Set element **Intention Tremor Left** to **Mild**

**Rule nose finger test left moderate**  
**When**  
    Element **Nose-finger test-left hand** equals to **Tremor with an amplitude < 5 cm**  
**Then**  
    Set element **"**Nose finger test left severity value**MAGNITUDE"** to 3  
    Set element **Intention Tremor Left** to **Moderate**

**Rule nose finger test left severe**  
**When**  
    ((  
        Element **Nose-finger test-left hand** equals to **Tremor with an amplitude > 5 cm**  
    )    **or** (  
        Element **Nose-finger test-left hand** equals to **Unable to perform 5 pointing movements**  
    ))  
**Then**  
    Set element **"**Nose finger test left severity value**MAGNITUDE"** to 4  
    Set element **Intention Tremor Left** to **Severe**

**Rule fast alternating hand movements right normal**  
**When**  
    Element **Fast alternating hand movements-right hand** equals to **Normal, no irregularities (performs <10s)**  
**Then**  
    Set element **Fast alternating hand movements-right hand** to **Fast alternating hand movements-right hand**  
    Set element **Dysdiadochokinesis Right** to **Normal**

**Rule fast alternating hand movements right mild**  
**When**  
    Element **Fast alternating hand movements-right hand** equals to **Slightly irregular (performs <10s)**  
**Then**  
    Set element **"**Fast alternating hand movements right severity value** MAGNITUDE"** to 2  
    Set element **Dysdiadochokinesis Right** to **Mild**

**Rule fast alternating hand movements right moderate**  
**When**  
    Element **Fast alternating hand movements-right hand** equals to **Clearly irregular, single movements difficult to distinguish or relevant interruptions, but performs <10s**  
**Then**  
    Set element **Dysdiadochokinesis Right** to **Moderate**  
    Set element **"**Fast alternating hand movements right severity value** MAGNITUDE"** to 3

**Rule fast alternating hand movements right severe**  
**When**  
    ((  
        Element **Fast alternating hand movements-right hand** equals to **Very irregular, single movements difficult to distinguish or relevant interruptions, performs >10s**  
    )    **or** (  
        Element **Fast alternating hand movements-right hand** equals to **Unable to complete 10 cycles**  
    ))  
**Then**  
    Set element **"**Fast alternating hand movements right severity value** MAGNITUDE"** to 4  
    Set element **Dysdiadochokinesis Right** to **Severe**

**Rule fast alternating hand movements left normal**  
**When**  
    Element **Fast alternating hand movements-left hand** equals to **Normal, no irregularities (performs <10s)**  
**Then**  
    Set element **Dysdiadochokinesis Left** to **Normal**  
    Set element **Fast alternating hand movements-left hand** to **Fast alternating hand movements-left hand**

**Rule fast alternating hand movements left mild**  
**When**  
    Element **Fast alternating hand movements-left hand** equals to **Slightly irregular (performs <10s)**  
**Then**  
    Set element **"**Fast alternating hand movements left severity value**MAGNITUDE"** to 2  
    Set element **Dysdiadochokinesis Left** to **Mild**

**Rule fast alternating hand movements left moderate**  
**When**  
    Element **Fast alternating hand movements-left hand** equals to **Clearly irregular, single movements difficult to distinguish or relevant interruptions, but performs <10s**  
**Then**  
    Set element **"**Fast alternating hand movements left severity value**MAGNITUDE"** to 3  
    Set element **Dysdiadochokinesis Left** to **Moderate**

**Rule fast alternating hand movements left severe**  
**When**  
    ((  
        Element **Fast alternating hand movements-left hand** equals to **Very irregular, single movements difficult to distinguish or relevant interruptions, performs >10s**  
    )    **or** (  
        Element **Fast alternating hand movements-left hand** equals to **Unable to complete 10 cycles**  
    ))  
**Then**  
    Set element **"**Fast alternating hand movements left severity value**MAGNITUDE"** to 4  
    Set element **Dysdiadochokinesis Left** to **Severe**

**Rule heel shin slide right normal**  
**When**  
    Element **Heel-shin slide-right hand** equals to **Normal**  
**Then**  
    Set element **Heel-shin slide-right hand** to **Nose-finger test-right hand**  
    Set element **Lower Limb Dysmetria Right** to **Normal**

**Rule heel shin slide right mild**  
**When**  
    Element **Heel-shin slide-right hand** equals to **Slightly abnormal, contact to shin maintained**  
**Then**  
    Set element **"**Heel-shin slide right severtiy value**MAGNITUDE"** to 2  
    Set element **Lower Limb Dysmetria Right** to **Mild**

**Rule heel shin slide right moderate**  
**When**  
    Element **Heel-shin slide-right hand** equals to **Clearly abnormal, goes off shin up to 3 times during 3 cycles**  
**Then**  
    Set element **"**Heel-shin slide right severtiy value**MAGNITUDE"** to 3  
    Set element **Lower Limb Dysmetria Right** to **Moderate**

**Rule heel shin slide right severe**  
**When**  
    ((  
        Element **Heel-shin slide-right hand** equals to **Severely abnormal, goes off shin 4 or more times during 3 cycles**  
    )    **or** (  
        Element **Heel-shin slide-right hand** equals to **Unable to perform the task**  
    ))  
**Then**  
    Set element **"**Heel-shin slide right severtiy value**MAGNITUDE"** to 4  
    Set element **Lower Limb Dysmetria Right** to **Severe**

**Rule heel shin slide left normal**  
**When**  
    Element **Heel-shin slide-left hand** equals to **Normal**  
**Then**  
    Set element **Heel-shin slide-left hand** to **Heel-shin slide-left hand**  
    Set element **Lower Limb Dysmetria Left** to **Normal**

**Rule heel shin slide left mild**  
**When**  
    Element **Heel-shin slide-left hand** equals to **Slightly abnormal, contact to shin maintained**  
**Then**  
    Set element **"**Heel-shin slide left severity value**MAGNITUDE"** to 2  
    Set element **Lower Limb Dysmetria Left** to **Mild**

**Rule heel shin slide left moderate**  
**When**  
    Element **Heel-shin slide-left hand** equals to **Clearly abnormal, goes off shin up to 3 times during 3 cycles**  
**Then**  
    Set element **"**Heel-shin slide left severity value**MAGNITUDE"** to 3  
    Set element **Lower Limb Dysmetria Left** to **Moderate**

**Rule heel shin slide left severe**  
**When**  
    ((  
        Element **Heel-shin slide-left hand** equals to **Severely abnormal, goes off shin 4 or more times during 3 cycles**  
    )    **or** (  
        Element **Heel-shin slide-left hand** equals to **Unable to perform the task**  
    ))  
**Then**  
    Set element **"**Heel-shin slide left severity value**MAGNITUDE"** to 4  
    Set element **Lower Limb Dysmetria Left** to **Severe**

**Rule Midline and Gait**  
**When**  
    Element **Gait severity value** is greater than or equals to **Stance severity value**  
    Element **Gait severity value** is greater than or equals to **Sitting severity value**  
**Then**  
    Set element **Midline severity value** to **Gait severity value**

**Rule Midline and Stance**  
**When**  
    Element **Stance severity value** is greater than **Midline severity value**  
**Then**  
    Set element **Midline severity value** to **Stance severity value**

**Rule Midline and Sitting**  
**When**  
    Element **Sitting severity value** is greater than **Midline severity value**  
**Then**  
    Set element **Midline severity value** to **Sitting severity value**

**Rule Midline normal**  
**When**  
    ((  
        Element **Midline severity value** equals to **0**  
    )    **or** (  
        Element **Sara Total Score** is less than **3**   
    ))  
**Then**  
    Set element **Midline Ataxia** to **Normal**

**Rule Midline Borderline**  
**When**  
    Element **Sara Total Score** is greater than or equals to **3**   
    Element **Midline severity value** equals to **1**  
**Then**  
    Set element **Midline Ataxia** to **Borderline**

**Rule Midline Mild**  
**When**  
    Element **Sara Total Score** is greater than or equals to **3**   
    Element **Midline severity value** equals to **2**  
**Then**  
    Set element **Midline Ataxia** to **Mild**

**Rule Midline moderate**  
**When**  
    Element **Sara Total Score** is greater than or equals to **3**   
    Element **Midline severity value** equals to **3**  
**Then**  
    Set element **Midline Ataxia** to **Moderate**

**Rule Midline severe**  
**When**  
    Element **Sara Total Score** is greater than or equals to **3**   
    Element **Midline severity value** equals to **4**  
**Then**  
    Set element **Midline Ataxia** to **Severe**

**Rule Midline Profound**  
**When**  
    Element **Sara Total Score** is greater than or equals to **3**   
    Element **Midline severity value** equals to **5**  
**Then**  
    Set element **Midline Ataxia** to **Profound**

**Rule Has Abasia yes**   
**When**  
    Element **Gait** equals to **Unable to walk, even supported**  
**Then**  
    Set element **Abasia** to **Yes**

**Rule Has Abasia no**  
**When**  
    Element **Gait** is not equal to **Unable to walk, even supported**  
**Then**  
    Set element **Abasia** to **No**

**Rule Has Astasia yes**  
**When**  
    Element **Stance** equals to **Unable to stand for >10 s even with constant support of one arm**  
**Then**  
    Set element **Astasia** to **Yes**

**Rule Has Astasia no**  
**When**  
    Element **Stance** is not equal to **Unable to stand for >10 s even with constant support of one arm**  
**Then**  
    Set element **Astasia** to **No**

**Rule Has Anarthria yes**  
**When**  
    Element **Speech Disturbance** equals to **Speech unintelligible / anarthria**  
**Then**  
    Set element **Anarthria** to **Yes**

**Rule Has Anarthria no**  
**When**  
    Element **Speech Disturbance** is not equal to **Speech unintelligible / anarthria**  
**Then**  
    Set element **Anarthria** to **No**

**Rule Cerebellar syndrome no**  
**When**  
    ((  
        Element **Counter** is less than or equals to **1**  
    )    **or** (  
        Element **Sara Total Score** is less than or equals to **1**   
    ))  
**Then**  
    Set element **Cerebellar Syndrome** to **No**

**Rule Cerebellar syndrome no significant**  
**When**  
    Element **Counter** is greater than **1**  
    Element **Sara Total Score** is greater than **1**   
    Element **Sara Total Score** is less than **3**   
**Then**  
    Set element **Cerebellar Syndrome** to **No Significant**

**Rule Cerebellar syndrome mild**  
**When**  
    Element **Counter** is greater than **1**  
    Element **Sara Total Score** is greater than or equals to **3**   
    Element **Sara Total Score** is less than or equals to **8**   
**Then**  
    Set element **Cerebellar Syndrome** to **Mild**

**Rule Cerebellar syndrome moderate**  
**When**  
    Element **Counter** is greater than **1**  
    Element **Sara Total Score** is greater than **8**   
    Element **Sara Total Score** is less than or equals to **15**   
**Then**  
    Set element **Cerebellar Syndrome** to **Moderate**

**Rule Cerebellar syndrome severe**  
**When**  
    Element **Counter** is greater than **1**  
    Element **Sara Total Score** is greater than **15**   
**Then**  
    Set element **Cerebellar Syndrome** to **Severe**

**Rule Appendicular right and finger chase right**  
**When**  
    Element **Appendicular right severity value** is less than **Finger chase right severity value**  
**Then**  
    Set element **Appendicular right severity value** to **Finger chase right severity value**

**Rule Appendicular right and nose finger test right**  
**When**  
    Element **Appendicular right severity value** is less than **Nose finger test right severity value**  
**Then**  
    Set element **Appendicular right severity value** to **Nose finger test right severity value**

**Rule Appendicular right and fast alternating hand movements right**   
**When**  
    Element **Appendicular right severity value** is less than **Fast alternating hand movements right severity value**   
**Then**  
    Set element **Appendicular right severity value** to **Fast alternating hand movements right severity value**

**Rule Appendicular right and heel shin slide right**  
**When**  
    Element **Appendicular right severity value** is less than **Heel-shin slide right severtiy value**  
**Then**  
    Set element **Appendicular right severity value** to **Heel-shin slide right severtiy value**

**Rule Appendicular right normal**   
**When**  
    ((  
        Element **Sara Total Score** is less than **3**   
    )    **or** (  
        Element **Appendicular right severity value** equals to **0**  
    ))  
**Then**  
    Set element **Appendicular Ataxia Right** to **Normal**

**Rule Appendicular right mild**   
**When**  
    Element **Sara Total Score** is greater than or equals to **3**   
    Element **Appendicular right severity value** equals to **2**  
**Then**  
    Set element **Appendicular Ataxia Right** to **Mild**

**Rule Appendicular right moderate**   
**When**  
    Element **Appendicular right severity value** equals to **3**  
    Element **Sara Total Score** is greater than or equals to **3**   
**Then**  
    Set element **Appendicular Ataxia Right** to **Moderate**

**Rule Appendicular right severe**   
**When**  
    Element **Sara Total Score** is greater than or equals to **3**   
    Element **Appendicular right severity value** equals to **4**  
**Then**  
    Set element **Appendicular Ataxia Right** to **Severe**

**Rule Appendicular left and finger chase left**   
**When**  
    Element **Appendicular left severity value** is less than **Finger chase left severity value**   
**Then**  
    Set element **Appendicular left severity value** to **Finger chase left severity value**

**Rule Appendicular left and nose finger test left**   
**When**  
    Element **Appendicular left severity value** is less than **Nose finger test left severity value**  
**Then**  
    Set element **Appendicular left severity value** to **Nose finger test left severity value**

**Rule Appendicular left and fast alternating hand movements left**   
**When**  
    Element **Appendicular left severity value** is less than **Fast alternating hand movements right severity value**   
**Then**  
    Set element **Appendicular left severity value** to **Fast alternating hand movements right severity value**

**Rule Appendicular left and heel shin slide left**   
**When**  
    Element **Appendicular left severity value** is less than **Heel-shin slide left severity value**  
**Then**  
    Set element **Appendicular left severity value** to **Heel-shin slide left severity value**

**Rule Appendicular left normal**   
**When**  
    ((  
        Element **Sara Total Score** is less than **3**   
    )    **or** (  
        Element **Appendicular left severity value** equals to **0**  
    ))  
**Then**  
    Set element **Appendicular Ataxia Left** to **Normal**

**Rule Appendicular left mild**   
**When**  
    Element **Sara Total Score** is greater than or equals to **3**   
    Element **Appendicular left severity value** equals to **2**  
**Then**  
    Set element **Appendicular Ataxia Left** to **Mild**

**Rule Appendicular left moderate**   
**When**  
    Element **Appendicular left severity value** equals to **3**  
    Element **Sara Total Score** is greater than or equals to **3**   
**Then**  
    Set element **Appendicular Ataxia Left** to **Moderate**

**Rule Appendicular left severe**   
**When**  
    Element **Sara Total Score** is greater than or equals to **3**   
    Element **Appendicular left severity value** equals to **4**  
**Then**  
    Set element **Appendicular Ataxia Left** to **Severe**
